# Pausing to swarm: locust intermittent motion is instrumental for swarming-related visual processing

**DOI:** 10.1098/rsbl.2023.0468

**Published:** 2024-02-21

**Authors:** Yossef Aidan, Itay Bleichman, Amir Ayali

**Affiliations:** ^1^ School of Zoology, Tel Aviv University, Tel Aviv 6997801, Israel; ^2^ Sagol School of Neuroscience, Tel Aviv University, Tel Aviv 6997801, Israel

**Keywords:** collective motion, intermittent, vision

## Abstract

Intermittent motion is prevalent in animal locomotion. Of special interest is the case of collective motion, in which social and environmental information must be processed in order to establish coordinated movement. We explored this nexus in locust, focusing on how intermittent motion interacts with swarming-related visual-based decision-making. Using a novel approach, we compared individual locust behaviour in response to continuously moving stimuli, with their response in semi-closed-loop conditions, in which the stimuli moved either in phase with the locust walking, or out of phase, i.e. only during the locust's pauses. Our findings clearly indicate the greater tendency of a locust to respond and ‘join the swarming motion’ when the visual stimuli were presented during its pauses. Hence, the current study strongly confirms previous indications of the dominant role of pauses in the collective motion-related decision-making of locusts. The presented insights contribute to a deeper general understanding of how intermittent motion contributes to group cohesion and coordination in animal swarms.

## 1. Introduction

Intermittent motion is a ubiquitous feature, instrumental in the locomotion of many animals [1,2]. It refers to a type of locomotion in which the animal alternates between periods of movement and periods of rest or pauses. While this phenomenon has often been explained in terms of conserving energy expenditure (e.g. [3–5], and see also [6]), alternative explanations have also been posited, including the role of intermittent motion in aspects of the animal's sensory biology [1]. The movement of animals utilizing intermittent, pause and go motion (run-and-tumble in bacteria; burst-and-coast in fish), can be described as comprising multiple discrete, repeated decisions regarding the initiation of movement as well as its direction [1]. Identifying and characterizing these decisions can contribute to a deeper understanding of the neurobiological and perceptual processes that underlie animal movement within their environment.

Of special interest is the case of intermittent motion in the context of animal collective motion [7]. Collective motion refers to the coordinated and synchronized movement patterns exhibited by groups of animals [8,9]. This phenomenon is observed in various species, including birds, fish, mammals and insects. In order to move in a harmonized manner, individuals within the coordinated group need to constantly acquire and process information regarding both their social and physical environments. This is critical in order to maintain the integrity and cohesion of the group and to be able to synchronize their movement to that of their conspecifics. Importantly, this rather demanding cognitive task takes place within an extremely complex and cluttered setting (i.e. the social and physical environment). Hence, collective motion involves complex interactions based on sensory perception, communication and individual behavioural adjustments within the group.

The above noted challenges may support the notion that, specifically in relation to collective motion, there are special advantages to intermittent motion. Indeed, intermittent motion of individuals has been widely demonstrated in the context of collective motion, from colonial bacteria [10–12], through schooling fish [13,14], to herds of mammals [15,16]. It has also been discussed in various theoretical models of collective motion (e.g. [17–20]). The advantages of intermittent motion of the individuals within a crowd pertain mostly to the sensory or sensory–motor integration domains rather than to motor control and energetics.

Nevertheless, although the intriguing phenomenon of intermittent motion has attracted much attention in the fields of behaviour and behavioural ecology, there have been very few attempts to directly relate this unique behaviour to aspects of sensory biology in general, and specifically to visual processing. In fact, in their recent review article Williams *et al*. [21], note that the sensory ecology of species is rarely incorporated into the field of collective behaviour, and that there is a great need to bridge these two areas of research and understand their interaction. These authors highlight the importance of exploring the sensory mechanisms used by organisms in order to extract the relevant signals and cues that serve in the control of collective behaviour-related decisions [21].

Intermittent, pause-and-go motion is also a dominant feature characterizing the behaviour of the swarms, or marching bands, of locust nymphs [22,23], which presents a quintessential example of collective motion in a natural system. Our recent report [24] provided initial insight into the cognitive abilities of locusts in the domain of decision-making and visual-based collective motion, supporting the use of locusts as a model for investigating sensory–motor integration and motion-related decision-making within the intricate swarm environment. In addition to the challenges noted by Bleichman *et al*. [24], Krongauz *et al*. [25] has recently discussed some other issues pertaining to swarming-related visual processing: namely, the problem of visual occlusions (see also [26]), as well as the basic challenges of assessing a locust neighbours' headings and speeds. The latter are of course further complicated by the subject locust's own motion relative to the environment as well as to its neighbours.

The current study thus constitutes a further effort to relate sensory processes, taking place at the level of the individual swarm member, to collective motion-related decisions. Moreover, this study is the first attempt to directly confirm the hypothesis that in locusts it is the sensory visual information perceived—collected and processed—specifically during the pauses that is crucial for the overall demonstrated collective behaviour.

**Figure 1.**
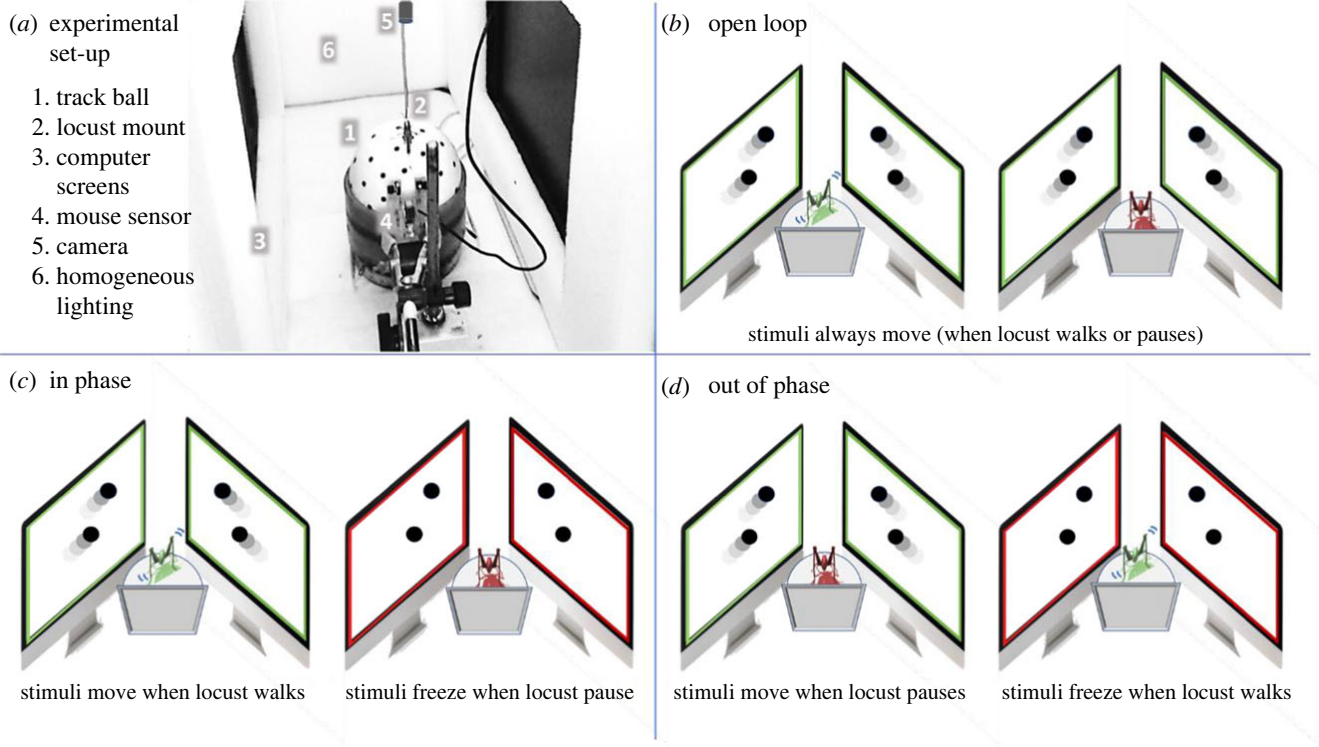
(*a*) A principal illustration of the overall experimental set-up depicting the different components. (*b–d*) Schematic illustrations of the three different types of experiments. (*b*) Open-loop experiment, i.e. the stimuli move in a *continuous* manner, irrespective of the locust's behaviour. (*c*) Semi-close-loop, *in phase*, i.e. the stimuli move in phase with the locust walking and freeze in phase with the locust pauses. (*d*) Semi-close-loop, *out of phase*, i.e. the stimuli move out of phase with the locust walking and freeze out of phase with the locust pauses. To better emphasize the different conditions in these illustrations, a colour code is used (for both the illustrated locusts and computer screens) where motion is depicted in green and stillness in red.

In this study, we employed a computer simulation to present carefully controlled moving stimuli to individual locusts. This simulation is aimed at replicating key features of the visual environment experienced by a locust within a marching locust swarm (i.e. the stimuli generated by the motion of swarm members surrounding the individual locust). We compared the walking kinematics of the locust in an open-loop experimental set-up, in which the stimuli move continuously independent of the locust intermittent behaviour (similar to that used in [24]), to its behaviour demonstrated under two semi-closed-loop conditions (figure 1), in which movement of the stimuli depends on the movement pattern of the locust: (1) in the *in-phase* experiments, the stimuli move in phase with the periods of the locust's walking and freeze when the locust pauses; and (2) in the *out-of-phase* experiments, the stimuli move out of phase with the locust's walking, i.e. they move only during the locust's pauses. A comparison of the findings from these somewhat unnatural experimental conditions, and specifically any differences between the behaviour under the two different semi-closed-loop conditions, would indicate a non-trivial process of stimuli processing and sensory–motor integration. Specific evidence of enhanced behavioural (swarming-related) response in the *out-of-phase experiment* would provide us with final validation for our hypothesis regarding the instrumental role of pauses in locust collective motion [22,23]. Indeed, our findings clearly indicated a greater tendency of the individual locust to respond (i.e. ‘join the swarm’) when exposed to the visual stimuli only during pauses (compared to only during walking or even continuously). Hence, the current study strongly confirms our previous contention of the central role of pauses, and intermittent motion in general, in collective motion-related decision-making.

## 2. Results and discussion

**Figure 2.**
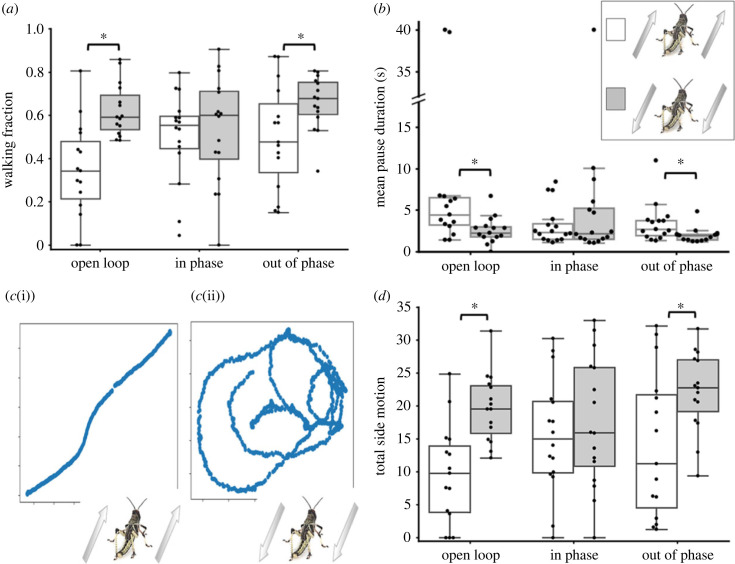
Locust walking kinematics in the three experimental conditions: open loop (i.e. continuously moving stimuli), in phase, and out of phase (see figure 1 and text for details). (*a*) Walking fraction. (*b*) Mean pause duration. (*c*) Two examples of the locusts' calculated walking trajectory as obtained from monitoring the movement of the trackball (the computed two-dimensional fictive path of 40 s are shown from two open loop experiments): (i) a locust overall sustaining forward motion in response to forward moving stimuli; and (ii) a locust demonstrating increased side motion, i.e. practically continuous turning behaviour, in response to backwards moving stimuli. See text for details. (*d*) Total side motion in the three experimental conditions. Open and shaded bars denote data obtained in experiments with the two types of stimuli (respectively): moving forward, versus moving backwards, opposite the heading direction of the locust, as depicted in the inset at the top right. Statistical significance of the difference in the values obtained within each experiment for forward versus backwards moving stimuli are indicated (asterisks denote *p* < 0.05, Mann–Whitney test, *n* = 15).

We recently established that locusts, subjected to experimental conditions and visual stimuli akin to those employed in the current study, exhibit walking kinematics that strongly suggest a propensity to synchronize their movement with the visual cues provided, i.e. moving black dots on a white screen, intended to mimic swarming-related visual stimuli ([24] and fig. 2 therein). When exposed to continuously moving stimuli on the two screens under open-loop conditions, locusts consistently showed a preference for walking in their (tethered) heading direction. However, when presented with stimuli moving in the opposite direction to their heading the locusts' behaviour was characterized by a higher walking fraction and shorter mean pause duration (figure 2*a,b*; open loop): this behaviour suggests an active effort to reverse their direction, likely in an attempt to rejoin the swarm. This was best manifested in a substantial difference in the relative proportion of lateral motion (turning) between the two types of stimuli (determined based on the direction of the trackball rotation; figure 2*c*,*d*; open loop). Note the straightness of the path shown in figure 2*c*(i), representing overall forward motion. In contrast, in the example shown in figure 2*c*(ii) the combination of the consistent backwards movement of the stimuli (in the open loop condition) and the fact that the locust cannot change its heading (due to the tether) result in a response that comprise continuous turning (the direction of the turn is random and not consistent between experiments; see [24] fig. 3*b* therein).

Figure 2 provides a comprehensive summary of the locusts’ response to the presented visual stimuli across the different experimental conditions: the open-loop and the two opposite semi-closed-loop conditions. Notable differences are evident in relation to the specific kinematic parameters associated with intermittent motion, namely the fraction of time spent walking (walking fraction) and the average pause duration (depicted in figure 2*a* and *b* respectively). Distinct differences between the two semi-closed loop conditions are manifested in the stimuli-direction-dependent response. As noted, in the open loop conditions, statistically significant differences in the kinematic parameter values are observed based on the direction of the stimuli. These differences are retained and are very pronounced in the out-of-phase experiments, while they become indistinct in the in-phase condition. Similarly, a comparable trend emerges when examining the locusts' inclination to turn or change direction. This tendency serves as a crucial indicator of the locusts’ decision-making process regarding their walking direction (figure 2*c*). Upon comparing the total side motion across the different experimental conditions, once again, significant stimuli-direction-dependent differences are evident in the open loop experiment. These distinct patterns also materialize in the out-of-phase experiment, but are lost in the in-phase experiment (figure 2*d*).

It should be noted that these data were collected by presenting the different stimuli (across the three different experiments) to distinct individual locusts. electronic supplementary material, figure S1 provides additional verification of the aforementioned findings through the results obtained when employing a somewhat different experimental design, in which each individual locust was presented with all the different stimulus types in a consecutive, random order (on the pivotal condition that prompts the locusts to turn and reverse their direction). These results support the overall findings as described above.

The current findings hence offer a direct and clear indication of swarming-related decisions being based on (visual) sensory information acquired by the animal mostly during pauses. In their pioneering investigation of the walking kinematics of single locusts in an experimental arena, Bazazzi *et al*. [27], already demonstrated a correlation between direction shifts and pause duration, thus indicating decision-making during pauses. Extending these results to locust groups, Ariel *et al*. [22] showed that while locusts terminated walks at random and the process has no memory component (a Markov process), pause times revealed a power-law distribution, suggesting an underlying information processing process involving memory (see review summary in [23]). All such studies to date, however, have been correlative in essence, as they were predominantly, or even exclusively, based on statistical quantification of the insects' walking kinematics (while alone or in a group). Our current reported findings suggest that intermittent locomotion plays a crucial role in the emergence of order in swarms of marching locust nymphs, and confirm its importance to the formation and maintenance of the swarm, as every time an animal starts moving following a pause it makes a small decision as to whether or not (and in what direction) to join the crowd. These findings constitute a first clear and direct indication of the instrumental role of intermittent motion in locust collective motion.

Visual motion information is crucial in behavioural control in a wide range of contexts, from navigation to foraging, from escaping predators to prey pursuit. In all these behaviours, moving through the natural environment presents a prominent challenge. In collective motion, the additional complexity of the social environment and the need to coordinate and synchronize with it adds an extra obstacle. Hence, although intermittent motion has been described as taking place during some of the above behavioural tasks (e.g. prey pursuit; [28]), it is during collective motion that pauses probably play a highly critical role. An additional point, already discussed by Harpaz *et al*. [14] in the context of the behaviour of fish, and which is also true for locusts, relates to the fact that neighbouring individuals in the swarm do not synchronize their pauses (see also [29]). This strongly supports a role for pauses in information processing by the individual (rather than supporting any general efficiency or energy-related models).

Finally, to the best of our knowledge, intermittent motion as a dominant feature of locomotion has thus far not been incorporated into bio-inspired technology, specifically in the growing field of insect-inspired robotics [30–34], particularly, in the prominent swarm-robotics technology. This is expected to be of special importance in the recently reported emerging field of hybrid insect–robotic swarms [35]. The current work thus contributes important insights, providing the means with which to embark upon these and other similarly important and promising interdisciplinary endeavours.

## 3. Methods

### (a) Animals

All trials were conducted using last (Vth) instar nymphs of desert locusts, *Schistocerca gregaria*, from our colony at the School of Zoology, Tel Aviv University. Only fully intact insects were used. Insects were deprived of food on the morning of the experiments in order to increase their general tendency for locomotion.

### (b) Experimental set-up

The experimental set-up largely followed that described in detail in [24]. Briefly, individual locusts were tethered in a walking posture above an airflow-suspended Styrofoam trackball, patterned in black over white to facilitate movement tracking and illuminated from above with LED lights. Controlled visual stimuli were presented on two parallel LCD screens, positioned on either side of the locust, spaced 30 cm apart. The locust's behavioural responses (i.e. the movement of the trackball) were monitored by way of an optical mouse sensor attached to the trackball and a high-speed video camera, and analysed using FICTRAC [36], an open-source software library for reconstructing the fictive path of an animal walking on a patterned sphere. The experimental chamber was temperature-controlled at 28°C to ensure optimal conditions for the locusts.

### (c) Visual stimulation

Python 3.9, along with several libraries, including Pygame and Multiprocessing were used to construct the visual stimuli in the experiments. The core libraries used in this study were developed using object-oriented programming techniques, enabling easy modification if necessary. Each screen operated independently, allowing for a wide range of artificial environments and trials to be created.

The primary visual stimulus used was the random-dot kinematogram (RDK), commonly used in motion perception studies. The visual simulations were parameterized to have a 100% coherence level, an equal speed of approximately 5 cm s^−1^ for each dot, and a refresh rate of 120 frames per second. Three types of visual stimulation were used (see figure 1):

1. Open-loop: visual stimuli did not change in response to the locust's behavioural state
2. Semi-closed-loop, in phase: the stimuli moved during the locust's walking bouts and froze during its pausing bouts.
3. Semi-closed-loop, out of phase: the stimuli froze during the locust walking bouts and moved during its pausing bouts.

Each type of visual simulation was used in two different directions of motion, either aligned with the tethered locust's heading or opposite to its heading, in order to analyse the test locust's behaviour under various control systems and contexts.

### (d) Semi-closed-loop control systems

In order to present the (semi) closed-loop control systems, the optical mouse sensor was connected to an Arduino micro-controller via a USB-host shield to capture the locust's locomotion. The two windows and an Arduino sensor were separate processes that communicated with each other via a common variable transmitting a Boolean state conveying the motion state of the insect. To address the sensor's high sensitivity and the potential for excessive signal input, a threshold was set in the Arduino code. The sensor enabled the creation of feedback-controlled systems that simulated different types of insect visual environment interactions (simulated swarm behaviours). When the locust was moving, the induced rotation of the trackball was perceived by the Arduino sensor, which modified the properties of the visual stimulus displayed, in the context of the specific type of experiment.

### (e) Behavioural analysis

The effects of the different visual stimuli on the locust's behaviour in the different experiments was analysed using similar parameters to those described in [24], including fraction of time spent walking (walking fraction), average pause duration, total distance travelled, and total demonstrated side motion. Behavioural data were analysed with Python scripts using Matplotlib Numpy and Scipy packages. All the data were plotted using the Matplotlib Python package and statistical tests were performed using GraphPad Prism 10 (GraphPad Software, San Diego, CA, USA). *P*-values of 0.05 were deemed statistically significant.

## Data Availability

All data supporting the findings of this study are available in this article and its electronic supplementary material, information. Video recordings of the experiments are being used in our ongoing research and can be made available upon direct request to the corresponding author. Supplementary material is available online [37].
